# Automated VMAT planning for postoperative adjuvant treatment of advanced gastric cancer

**DOI:** 10.1186/s13014-018-1032-z

**Published:** 2018-04-23

**Authors:** Abdul Wahab M. Sharfo, Florian Stieler, Oskar Kupfer, Ben J. M. Heijmen, Maarten L. P. Dirkx, Sebastiaan Breedveld, Frederik Wenz, Frank Lohr, Judit Boda-Heggemann, Daniel Buergy

**Affiliations:** 1 Department of Radiation Oncology, Erasmus MC Cancer Institute, Groene Hilledijk 301, 3075 EA Rotterdam, The Netherlands; 2 Universitätsmedizin Mannheim, Medical Faculty Mannheim, Heidelberg University, Mannheim, Germany; 3 Unita Operativa di Radioterapia, Dipartimento di Oncologia, Az. Ospedaliero-Universitaria di Modena, Modena, Italy

**Keywords:** VMAT, Automated planning, NTCP, Gastric cancer

## Abstract

**Background:**

Postoperative/adjuvant radiotherapy of advanced gastric cancer involves a large planning target volume (PTV) with multi-concave shapes which presents a challenge for volumetric modulated arc therapy (VMAT) planning. This study investigates the advantages of automated VMAT planning for this site compared to manual VMAT planning by expert planners.

**Methods:**

For 20 gastric cancer patients in the postoperative/adjuvant setting, dual-arc VMAT plans were generated using fully automated multi-criterial treatment planning (autoVMAT), and compared to manually generated VMAT plans (manVMAT). Both automated and manual plans were created to deliver a median dose of 45 Gy to the PTV using identical planning and segmentation parameters. Plans were evaluated by two expert radiation oncologists for clinical acceptability. AutoVMAT and manVMAT plans were also compared based on dose-volume histogram (DVH) and predicted normal tissue complication probability (NTCP) analysis.

**Results:**

Both manVMAT and autoVMAT plans were considered clinically acceptable. Target coverage was similar (manVMAT: 96.6 ± 1.6%, autoVMAT: 97.4 ± 1.0%, *p* = 0.085). With autoVMAT, median kidney dose was reduced on average by > 25%; (for left kidney from 11.3 ± 2.1 Gy to 8.9 ± 3.5 Gy (*p* = 0.002); for right kidney from 9.2 ± 2.2 Gy to 6.1 ± 1.3 Gy (*p* <  0.001)). Median dose to the liver was lower as well (18.8 ± 2.3 Gy vs. 17.1 ± 3.6 Gy, *p* = 0.048). In addition, Dmax of the spinal cord was significantly reduced (38.3 ± 3.7 Gy vs. 31.6 ± 2.6 Gy, *p* <  0.001). Substantial improvements in dose conformity and integral dose were achieved with autoVMAT plans (4.2% and 9.1%, respectively; *p* <  0.001). Due to the better OAR sparing in the autoVMAT plans compared to manVMAT plans, the predicted NTCPs for the left and right kidney and the liver-PTV were significantly reduced by 11.3%, 12.8%, 7%, respectively (*p* ≤ 0.001). Delivery time and total number of monitor units were increased in autoVMAT plans (from 168 ± 19 s to 207 ± 26 s, *p* = 0.006) and (from 781 ± 168 MU to 1001 ± 134 MU, *p* = 0.003), respectively.

**Conclusions:**

For postoperative/adjuvant radiotherapy of advanced gastric cancer, involving a complex target shape, automated VMAT planning is feasible and can substantially reduce the dose to the kidneys and the liver, without compromising the target dose delivery.

**Electronic supplementary material:**

The online version of this article (10.1186/s13014-018-1032-z) contains supplementary material, which is available to authorized users.

## Background

In the Intergroup Study 0116 (INT-0116) study, adjuvant chemoradiotherapy (CRT) improved overall survival and progression-free survival in patients with gastric cancer compared to surgery alone [1, 2]. The benefit of combined modality treatment was later confirmed in Asian studies with D2-resected patients [3–5]. European trials in gastroesophageal junction cancers showed survival benefits compared to surgery with neoadjuvant CRT [6] or perioperative chemotherapy without radiotherapy [7, 8]. Randomized trials comparing adjuvant CRT to (perioperative) chemotherapy indicated clinical benefits for CRT in subgroups, but failed in showing a consistent benefit in European and Asian trials [3, 5, 9, 10]. Therefore, the role of adjuvant CRT in gastric carcinoma compared to other approaches, such as perioperative chemotherapy, is not yet clearly defined [9]. The use of modern radiotherapy approaches might improve the risk−/benefit ratio in favor of radiotherapy in gastric carcinoma. Recent data showed the feasibility of using intensity-modulated radiation therapy (IMRT) or volumetric modulated arc therapy (VMAT) for this purpose [3, 10–12]. Postoperative radiotherapy of advanced gastric cancer involves a large target volume with multi-concave shapes which presents a challenge for VMAT planning with a risk of protocol deviations. In a similar setting, radiotherapy for esophageal carcinoma indicated that the experience of the treatment planner may largely affect plan quality, as shown by large differences in plan quality among planners within one institute [13]. In such a setting, automated treatment planning might have several advantages, including facilitated central review in clinical trials as well as improvement of less experienced treatment planner’s performance.

Over the years, in-house developed as well as commercial algorithms have attempted to automate the trial-and-error process in order to create optimal plans, reduce user variability and improve the quality and efficiency of the resulting plans. Knowledge-based planning uses a model library of previously generated plans to predict new treatment plan parameters [14, 15], while multi-criterial optimization generates a set of Pareto-optimal plans [16, 17]. Erasmus-iCycle is an optimizer for fully automated multi-criterial beam profile optimization and beam angle selection for coplanar and non-coplanar IMRT, developed at the Erasmus MC Cancer Institute [18–22]. In combination with the Monaco treatment planning system (TPS), Erasmus-iCycle is currently used in clinical practice for IMRT and VMAT plan generation for prostate, head and neck, cervical and lung cancer patients [19–22]. Several studies on postoperative gastric cancer patients have dosimetrically evaluated different radiotherapy techniques [23–26]. To our knowledge, no study has been published considering the feasibility and advantages of automated treatment planning for this treatment site. In this study, we investigated to what extent automated treatment planning using Erasmus-iCycle results in improved VMAT plan quality for advanced gastric cancer patients compared to plan generation by an expert planner.

## Methods

### Patients

A total of 20 patients with advanced gastric cancer who received radiotherapy treatment were included in the study. Clinical details are shown in Additional file 1: Table S1. Written informed consent was obtained from all patients for anonymized usage of treatment planning data. Our study protocol for retrospective evaluation of automated planning using Erasmus-iCycle was approved by the ethics committee of Heidelberg University, Medical Faculty Mannheim (2016-806R-MA).

### Treatment plan generation

All manually and automatically generated dual-arc VMAT plans (manVMAT and autoVMAT, respectively) were prescribed to deliver a median dose of 45 Gy to the target in 25 fractions. The primary planning objective was achieving adequate PTV coverage while maximally sparing the organs at risk. All plans were generated for delivery at a VersaHD linear accelerator (Elekta AB, Stockholm, Sweden), equipped with an Agility multi-leaf collimator and a photon beam energy of 10 MV.

All manVMAT plans were generated by expert treatment planners with the Monaco TPS, version 5.11 (Elekta AB, Stockholm, Sweden) using template-based optimization cost-functions. The employed optimization template is set according to the current clinical guidelines used at the Medical University of Mannheim. For each patient, the optimization cost-functions’ parameters were iteratively tweaked to improve the dose distribution and achieve optimal target coverage while respecting OAR constraints. As explained below, also the final autoVMAT plans were generated with Monaco, for which we used the same software version. All Monte Carlo dose calculations in Monaco were performed using a 1% dose variance, and a dose grid resolution of 3 mm. A maximum of 140 control points per arc was allowed. Also the other segmentation settings were kept identical for manual and automated plan generation.

### Automated multi-criterial VMAT plan generation with Erasmus-iCycle/Monaco

The in-house developed Erasmus-iCycle/Monaco platform for fully automated multi-criteria plan generation [18–22] was configured to generate clinically deliverable VMAT plans for gastric cancer. For each of the study patients, Erasmus-iCycle was used to first automatically generate a *Pareto-optimal* plan with clinically favorable trade-offs between treatment objectives using a “*wish-list*” developed for gastric cancer (as detailed below in Table 1). Based on this Erasmus-iCycle plan, a patient-specific Monaco template was then created fully automatically, to be used in the Monaco TPS for automated generation of a deliverable dual-arc VMAT plan that mimicked the initial Erasmus-iCycle plan. The applied wish-list contains constraints to be strictly fulfilled, and clinical plan objectives with ascribed priorities to be met as closely as possible or superseded (see Table 1 for details). Three constraints were used to control the maximum dose in the PTV and the patient (i.e., the outline of the patient’s external surface including the PTV, delineated OARs and the unspecified tissues), as well as the dose conformity outside the PTV. In order to achieve a homogeneous and adequate PTV dose coverage, the highest priority objective was given to the PTV using the Logarithmic Tumor Control Probability function (LTCP) [27], followed by a shell around the PTV to ensure a steep gradient outside the PTV (priority 2). In line with the clinical practice, minimizing the mean dose in the kidneys was the organ-at-risk (OAR) objective with highest priority (priority 3), followed by the mean dose in the liver (priority 4). Subsequently, equivalent uniform dose (EUD) objectives with volume effect parameters (k = 6 and 12) [28], focusing on reduction of the midrange and high dose in the heart and spinal cord were used (priorities 5 and 6), respectively. To control the dose conformity and entrance dose, a shell at 18 mm from the PTV, as well as a skin ring of 21 mm wide from the body contour towards the patient’s internal were defined (priority 7). Additionally, dose-volume objectives for the kidneys, liver, and the lungs were used with lower priorities.

**Table 1:** Applied wish-list for automatic VMAT plan generation for gastric cancer patients

Constraints				
Volume		Type	Limit	
PTV		Maximum	105% of *D*^*p*^	
PTV Shell 39 mm		Maximum	50% of *D*^*p*^	
Patient		Maximum	105% of *D*^*p*^	
Objectives				
Priority	Volume	Type	Goal	Parameters
1	PTV	↓ LTCP	0.4	*D*^*p*^=45 Gy,α = 4
2	PTV Shell 3 mm	↓ Maximum	90% of *D*^*p*^	
3	Left and Right Kidney	↓ Mean	8 Gy	
4	Liver	↓ Mean	15 Gy	
5	Heart	↓ EUD	15 Gy	k = 6
6	Spinal Cord	↓ EUD	25 Gy	k = 12
7	PTV Shell 18 mm	↓ Maximum	40% of *D*^*p*^	
	Skin Ring	↓ Maximum	25% of *D*^*p*^	21 mm
8	Left and Right Kidney	↓ Volume-Dose	25%	12 Gy
	Liver	↓ Volume-Dose	30%	24 Gy
9	Left and Right Lung	↓ Volume-Dose	50%	20 Gy

Abbreviations: *D*^*p*^ prescribed dose, *LTCP* Logarithmic Tumor Control Probability, *α* cell sensitivity parameter to achieve adequate target coverage, *EUD* Equivalent Uniform Dose, *k* volume parameter

### Plan evaluation and comparison

All plans were evaluated by expert radiation oncologists (FL, JBH, and DB) for clinical acceptability. For fair dosimetric plan comparisons, all manVMAT and autoVMAT plans were first normalized to obtain equal median PTV dose (i.e., D_50%_ = 45 Gy). In accordance with the International Commission on Radiation Units and Measurements Report No. 83, near-minimum and near-maximum doses (D_98%_ and D_2%_, respectively) in the PTV were evaluated, from which the homogeneity index (HI = (D_2%_ - D_98%_)/D_50%_) was computed. Additionally, dose conformity was estimated by calculating the conformity index (CI = (TV_RI_)^2^/(TV*V_RI_)), i.e., ratio of the target volume covered by the reference isodose level (TV_RI_) to the target volume (TV) and volume of the reference isodose (V_RI_). Quantitative analyses of OAR doses included D_mean_, D_30%_, D_50%_, D_60%_ in the kidneys and the liver-PTV, mean and maximum doses (D_mean_, and D_max_) in the spinal cord and the heart, volumes of the kidneys receiving more than 12 Gy (V_12Gy_) and 20 Gy (V_20Gy_) [29], volumes of the liver-PTV receiving more than 24 Gy (V_24Gy_) and 30 Gy (V_30Gy_), and D_mean_ in the lungs and volumes of the lungs receiving more than 5 Gy (V_5Gy_) and 20 Gy (V_20Gy_) [30]. In addition, integral patient doses were evaluated by assessing patient D_mean_ and volumes receiving V_5Gy_, V_11.25Gy_ and V_22.5Gy_. Estimated treatment delivery time and total number of monitor units (MUs) for each plan were also quantified.

From the DVHs, normal tissue complication probabilities (NTCPs) for the kidneys and the liver were estimated using the Lyman-Burman-Kutcher NTCP model for late effects, considering tolerance dose TD_50/5_ = 12 Gy, *n* = 0.70, *m* = 0.26 for the kidneys [29], and TD_50/5_ = 30 Gy, *n* = 0.32, *m* = 0.15 for liver failure [31], where TD_50/5_ refers to the dose to the whole organ which lead to complication in 50% of the population at 5 years, *m* relates to the steepness of the dose-response curve, and *n* represents the volume effect in the LKB model. For this purpose, all plans were first normalized to 1.5 Gy per fraction using α/β = 2.5 Gy.

Differences in dosimetric parameters between manVMAT and autoVMAT plans were analyzed using SPSS software v.21 (SPSS, Inc., Chicago, USA) and presented as the mean ± 1 standard deviation. Paired two-sided Wilcoxon Signed-rank tests were performed to assess statistical significance of observed differences, considering *p* <  0.05 statistically significant.

## Results

### Target volume dosimetric evaluations

**Fig. 1:**
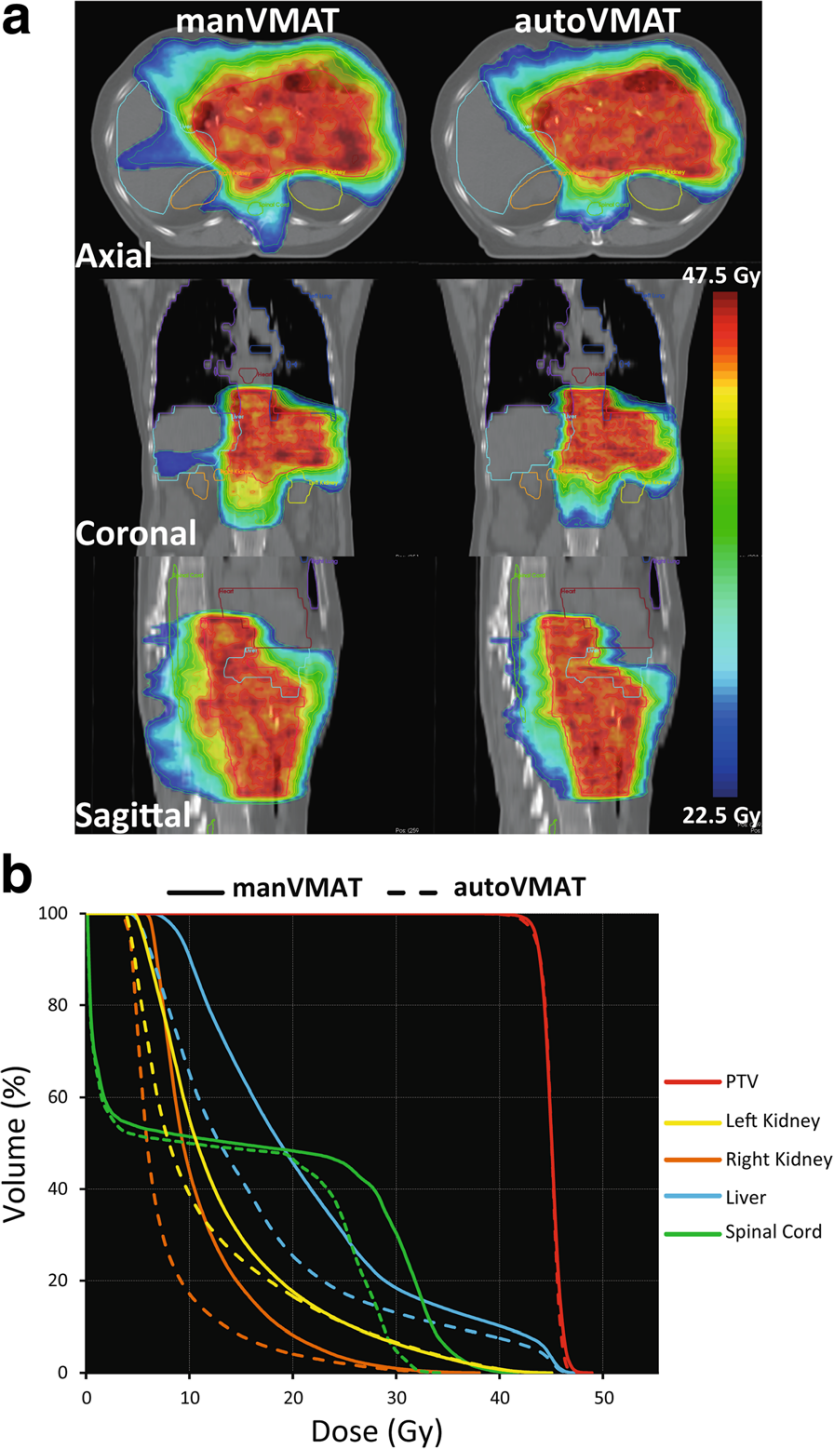
**a** Comparison of dose distributions for the manVMAT (left) and autoVMAT plans (right) for patient 8 on the axial, coronal and sagittal planes, (**b**) dose volume histograms for the manVMAT (solid lines) and the autoVMAT (dashed lines) plans of this patient

All automatically and manually generated VMAT plans were clinically acceptable and achieved adequate target coverage. Clinical acceptability of the plans was evaluated by radiation oncologists with experience in gastric cancer treatment (JBH, FL and DB). Differences between the autoVMAT and manVMAT plans in V_95%_ or D_98%_ were not statistically significant (Table 2). However, autoVMAT plans exhibited a significantly lower near-maximum dose in the PTV, resulting in a better target dose homogeneity (0.09 ± 0.01 vs. 0.10 ± 0.02 (*p* = 0.003)). For autoVMAT, the dose conformity was significantly improved as well (0.91 ± 0.02 vs. 0.88 ± 0.03 (*p* <  0.001)). As an example, Fig. 1a shows the resulting dose distributions from the autoVMAT and manVMAT plans for patient 8. As is evident from this figure and the corresponding dose-volume histograms in Fig. 1b, AutoVMAT resulted in favorable dose conformity and better OAR sparing.

**Table 2:** Comparison of dosimetric parameters between autoVMAT and manVMAT plans. Population mean values for the 20 study patients and corresponding standard deviations are reported

Structure	Parameter	autoVMAT	manVMAT – autoVMAT	
		Mean ± SD	Mean diff. ± SD	*p*-value
PTV	V_95%_ (%)	97.4 ± 1.0	- 0.8 ± 1.7	0.085
	D_98%_ (Gy)	42.5 ± 0.5	- 0.2 ± 0.7	0.14
	D_2%_ (Gy)	46.3 ± 0.1	0.5 ± 0.2	< 0.001
	HI	0.09 ± 0.01	0.02 ± 0.02	0.003
	CI	0.91 ± 0.02	- 0.04 ± 0.03	< 0.001
Left Kidney	D_mean_ (Gy)	12.7 ± 4.3	1.8 ± 1.6	0.001
	D_30%_ (Gy)	14.9 ± 8.1	2.1 ± 3.3	0.013
	D_50%_ (Gy)	8.9 ± 3.5	2.4 ± 2.2	0.002
	D_60%_ (Gy)	7.3 ± 1.9	2.4 ± 1.4	< 0.001
	V_12Gy_ (%)	32.2 ± 14.0	11.7 ± 7.7	< 0.001
	V_20Gy_ (%)	19.2 ± 13.2	1.0 ± 3.9	0.117
	NTCP (%)	45.1 ± 35.8	11.3 ± 10.9	0.001
Right Kidney	D_mean_ (Gy)	7.5 ± 1.7	3.0 ± 1.2	< 0.001
	D_30%_ (Gy)	7.8 ± 1.7	4.1 ± 1.6	< 0.001
	D_50%_ (Gy)	6.1 ± 1.3	3.1 ± 1.3	< 0.001
	D_60%_ (Gy)	5.5 ± 1.2	2.7 ± 1.1	< 0.001
	V_12Gy_ (%)	12.4 ± 4.8	18.9 ± 8.4	< 0.001
	V_20Gy_ (%)	3.8 ± 2.8	3.0 ± 2.8	0.001
	NTCP (%)	4.2 ± 3.8	12.8 ± 7.2	< 0.001
Liver - PTV	D_mean_ (Gy)	18.6 ± 3.3	1.8 ± 2.8	0.02
	D_30%_ (Gy)	21.9 ± 4.1	2.8 ± 3.2	0.003
	D_50%_ (Gy)	17.1 ± 3.6	1.7 ± 3.3	0.048
	D_60%_ (Gy)	15.1 ± 3.4	1.2 ± 3.3	0.14
	V_24Gy_ (%)	25.4 ± 11.6	7.1 ± 8.8	0.004
	V_30Gy_ (%)	12.9 ± 6.0	4.7 ± 4.6	0.001
	NTCP (%)	31.1 ± 23.2	7.0 ± 7.0	0.001
Heart	D_mean_ (Gy)	12.7 ± 4.7	0.7 ± 1.8	0.117
	D_max_ (Gy)	46.8 ± 0.6	0.8 ± 1.2	0.009
Spinal cord	D_mean_ (Gy)	13.9 ± 2.6	2.9 ± 1.5	< 0.001
	D_max_ (Gy)	31.6 ± 2.6	6.7 ± 3.2	< 0.001
Left Lung	D_mean_ (Gy)	7.3 ± 3.2	0.6 ± 0.3	< 0.001
	V_5Gy_ (%)	32.7 ± 15.6	1.4 ± 1.0	< 0.001
	V_20Gy_ (%)	14.0 ± 7.2	2.2 ± 1.8	< 0.001
Right Lung	D_mean_ (Gy)	5.4 ± 3.3	0.3 ± 0.5	0.013
	V_5Gy_ (%)	31.2 ± 16.8	1.1 ± 2.1	0.01
	V_20Gy_ (%)	7.0 ± 7.6	0.8 ± 1.8	0.078
Patient	D_mean_ (Gy)	9.4 ± 1.9	0.5 ± 0.3	< 0.001
	V_5Gy_ (%)	40.2 ± 8.9	1.6 ± 0.8	< 0.001
	V_11.25Gy_ (%)	30.0 ± 6.6	1.8 ± 1.7	0.001
	V_22.5Gy_ (%)	16.2 ± 3.3	1.5 ± 1.1	< 0.001

Abbreviations: *HI* homogeneity index, *CI* conformity index, *NTCP* normal tissue complication probability

### Organs at risk dosimetric evaluations

**Fig. 2:**
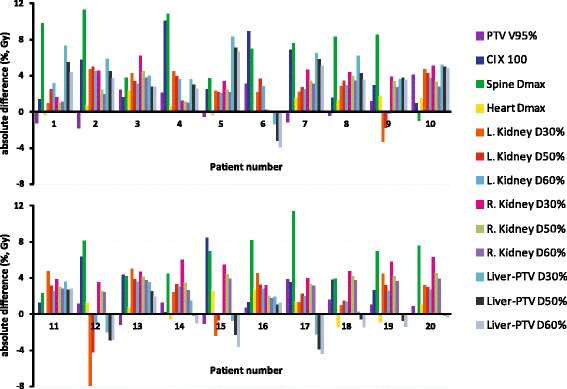
Differences in dosimetric plan parameters between autoVMAT and manVMAT plans for each of the 20 study patients. Positive values are in favor of the autoVMAT plans

Figure 2 shows the observed absolute differences in dosimetric parameters for each of the study patients. Overall, autoVMAT plans had more favorable dose distributions, resulting in reduced dose delivery to the kidneys, liver, spinal cord, heart and lungs, without deteriorating the PTV dose coverage (Table 2 and Fig. 2). Median doses (D_50%_) to the left kidney, right kidney, and liver-PTV were significantly reduced by 28% (from 11.3 ± 2.1 Gy to 8.9 ± 3.5 Gy, *p* = 0.002), 39% (from 9.2 ± 2.2 Gy to 6.1 ± 1.3 Gy, *p* <  0.001), and 11% (from 18.8 ± 2.3 Gy to 17.1 ± 3.6 Gy, *p* = 0.048), respectively. The V_20Gy_ for the left and right kidney with autoVMAT were lower than those for the manVMAT plans (Table 2). In addition, the V_30Gy_ for the liver-PTV was significantly decreased with autoVMAT plans by 36% (from 17.7 ± 6.2% to 12.9 ± 6.0%, *p* = 0.001). The maximum dose in the spinal cord was on average reduced by 6.7 ± 3.2 Gy (*p* <  0.001) with autoVMAT. Furthermore, the integral dose in the patient and the dose conformity were also significantly better with autoVMAT (Table 2).

### NTCPs evaluations

**Fig. 3:**
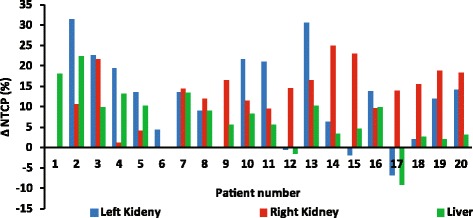
Differences in the predicted normal tissue complication probabilities (NTCP) between autoVMAT and manVMAT plans for the 20 study patients. Positive values are in favor of the autoVMAT plans

The published sets of parameters used for calculations of NTCPs for the kidneys and liver-PTV resulted in overall lower probability of late complications with autoVMAT compared to manVMAT plans. In 16/20 patients, the predicted NTCPs were lowest with autoVMAT for the left kidney, whereas all 20 patients had lower predicted NTCPs with autoVMAT for the right kidney (Fig. 3). For 17/20 patients, the NTCP predictions for liver-PTV were lower for the autoVMAT plans. The average NTCP values resulting from autoVMAT, given in Table 2, were significantly reduced by 11.3%, 12.8% and 7% for the left and right kidney and the liver-PTV, respectively.

### Planning and treatment delivery times

All manVMAT plans in this study were generated using template-based manual planning. Hands-on tweaking time of the optimization cost functions was on average 30 min (range 5-60 min). The optimization and dose calculation of autoVMAT plans in Erasmus-iCycle/Monaco was fully automated and therefore did not require any hands-on time.

Compared to manVMAT plans a significantly more MUs were required for delivery of the autoVMAT plans (1001 ± 134 MU vs. 781 ± 168 MU, *p* = 0.003), resulting in a prolonged (estimated) treatment delivery time (207 ± 26 s vs. 168 ± 19 s, *p* = 0.006).

## Discussion

Gastric cancer is the third leading cause of cancer death in both sexes with yearly 723,000 deaths worldwide [32]. Protocol deviations can reduce clinical efficacy of radiotherapy in complex treatment geometries, like gastric carcinoma. In the centralized review of the INT-0116 study, minor or major protocol deviations were observed in 35% of patients [2]. Even after correction of errors, major protocol deviations were still found in 6.5% of the plans that were actually irradiated [1, 2]. The clinical impact of protocol deviations in INT-0116 has not been reported. However, in other complex geometries it was shown that protocol violations during radiation therapy were correlated with reduced overall survival [33]. Modern radiotherapy approaches such as IMRT and VMAT have several dosimetric advantages but they are associated with increased complexity [9, 34–36]. Very high protocol deviations are therefore possible; for example, RTOG 0529 showed 81% protocol deviations at first plan review [37]. Albeit in small numbers, RTOG 0022 showed local failure rates of 50% (2/4) vs. 6% (3/49) in oropharyngeal cancer patients treated with and without protocol deviations, respectively [38]. Protocol deviations may, amongst other things, be caused by inaccurate delineation or sub-optimal treatment planning. For gastric cancer, the percentage of these deviations is unknown. In a recent report on RTOG 0933 by Gondi et al. [39], unacceptable radiotherapy protocol deviations were observed in 25% of cases at a rapid review process: 11/21 cases had contouring deviations, 5/21 cases had unacceptable planning deviations and 5/21 had unacceptable deviations of contouring and planning. Also in a recent study of Habraken et al. [40] on hepatocellular carcinoma patients, it was demonstrated that pretreatment plan review was important to reduce unacceptable protocol deviations. Additionally, they demonstrated that automated treatment planning could be used to identify sub-optimal treatment plans. This might be especially relevant in trials in which low numbers of patients are eligible or small numbers of patients are treated in a participating hospital.

Over the past years, several studies have investigated the advantages of automated treatment planning compared to manual planning using in-house developed or commercial algorithms [18–22, 41–43]. Treatment planning with Erasmus-iCycle/Monaco is fully automated, and it has been successfully validated for clinical use in several treatment sites including prostate [19], head-and-neck [20], cervix [21], and lung [22]. Knowledge-based planning using a model library of previously generated plans to predict treatment plan parameters for a new patient was configured and tested in pelvic anatomy [41], lung [42], and esophageal cancer [43].

Numerous dosimetric studied have evaluated different radiotherapy techniques for gastric cancer by comparing IMRT, VMAT, helical tomotherapy, 3DCRT and proton therapy [23–26]. Until now, no study has been published showing the possibility/advantages of automated treatment planning for postoperative gastric cancer patients. In this study Erasmus-iCycle was used to automatically generate VMAT plans for gastric cancer patients. For this site, manual treatment optimization is a large challenge, even for an expert planner, due to the multi-concave shape, the extent of the target volume, and the close proximity to many radiosensitive organs (i.e., kidneys, liver, heart, and spinal cord). Another difficulty in postoperative radiotherapy in gastric cancer patients is that radiation tolerance doses of OARs are relatively low. Compared to the manVMAT plans, generated by an expert planner, plan quality was significantly better for the autoVMAT plans, only requiring slightly longer treatment delivery times. Specifically, dose conformity and sparing of organs at risk were improved, while the clinical importance of the observed longer treatment delivery time and the increased MUs is considered to be low. As a result of higher modulation, the number of MUs was significantly higher in the autoVMAT than in manVMAT plans, this could lead to challenges in radiation delivery. The integral dose was significantly reduced in autoVMAT plans, this may be beneficial specially for young patients to avoid secondary tumors, and most notably showed significantly reduced NTCP for liver and both kidneys, although it is not yet clear to what extent the observed dosimetric advantages of autoVMAT vs. an experienced planner will really translate into a clinical benefit. Automated treatment planning can improve the efficiency of the treatment planning process and reduce its user dependency, this in turns might lead to more consistent and uniform outcomes in treatment planning studies and clinical trials. Apart from the improved plan quality and reduced predicted complications, automated planning also eliminated the planning hands-on time required for planning.

## Conclusion

Automated treatment planning is of great value for complex treatment sites like for gastric cancer. Compared to manual planning by an expert planner, plan quality could be largely improved, while drastically reducing treatment planning workload.

## Additional file

Additional file 1: **Table S1.** Clinical details of patients with gastric carcinoma. (DOCX 15 kb)

## Abbreviations

autoVMAT: Fully automated volumetric modulated arc therapy (plans)
CI: Conformity index
CRT: Chemoradiotherapy
DVH: Dose-volume histogram
EUD: Equivalent uniform dose
HI: Homogeneity index
IMRT: Intensity-modulated radiation therapy
INT-0116: Intergroup Study 0116
LTCP: Logarithmic tumor control probability
manVMAT: Manually generated volumetric modulated arc therapy (plans)
MU: Monitor units
NTCP: Normal tissue complication probability
OAR: Organ at risk
PTV: Planning target volume
RTOG: Radiation Therapy Oncology Group
TPS: Treatment planning system
TV: Target volume
VMAT: Volumetric modulated arc therapy
