# Supplementary material for: Automated VMAT planning for postoperative adjuvant treatment of advanced gastric cancer
Source: Radiat Oncol. 2018 Apr 23;13:74. doi: 10.1186/s13014-018-1032-z (PMC5913894; doi:10.1186/s13014-018-1032-z)
Supplement: Supplementary file 1 — Table S1. Clinical details of patients with gastric carcinoma. (DOCX 15 kb) [file 13014_2018_1032_MOESM1_ESM.docx]

**Additional file 1**

**Table S1** Clinical details of patients with gastric carcinoma.

|  |  | **Number of patients** |
| --- | --- | --- |
| **Sex** | Female | 9 |
|  | Male | 11 |
| **Age at diagnosis** | < 50 | 4 |
|  | 50-55 | 5 |
|  | 56-60 | 5 |
|  | 61-65 | 2 |
|  | > 65 | 4 |
| **Staging** | | |
| T | cT1 | 2 |
|  | cT2a | 5 |
|  | cT2b | 4 |
|  | cT3 | 8 |
|  | cT4 | 1 |
| N | N0 | 2 |
|  | N1 | 13 |
|  | > N2 | 5 |
| M | M0 | 19 |
|  | M1 | 0 |
|  | Mx | 1 |
| **Resection margins** | R0 | 16 |
|  | R1 | 1 |
|  | Unknown | 3 |
| **Tumor site** | Cardia | 1 |
|  | Antrum | 2 |
|  | Corpus | 3 |
|  | Lesser curvature | 2 |
|  | Greater curvature | 1 |
|  | Diffuse | 5 |
|  | Unknown | 6 |
